# Critical Role of Intracellular RyR1 Calcium Release Channels in Skeletal Muscle Function and Disease

**DOI:** 10.3389/fphys.2015.00420

**Published:** 2016-01-12

**Authors:** Erick O. Hernández-Ochoa, Stephen J. P. Pratt, Richard M. Lovering, Martin F. Schneider

**Affiliations:** ^1^Department of Biochemistry and Molecular Biology, University of Maryland School of MedicineBaltimore, MD, USA; ^2^Department of Orthopaedics, University of Maryland School of MedicineBaltimore, MD, USA

**Keywords:** skeletal muscle, excitation-contraction coupling, sarcolemma, sarcoplasmic reticulum, Ca^2+^ release channel, ryanodine receptor type 1, RyR1-related mutations, RyR1 dysfunction and disease

## Abstract

The skeletal muscle Ca^2+^ release channel, also known as ryanodine receptor type 1 (RyR1), is the largest ion channel protein known and is crucial for effective skeletal muscle contractile activation. RyR1 function is controlled by Ca_v_1.1, a voltage gated Ca^2+^ channel that works mainly as a voltage sensor for RyR1 activity during skeletal muscle contraction and is also fine-tuned by Ca^2+^, several intracellular compounds (e.g., ATP), and modulatory proteins (e.g., calmodulin). Dominant and recessive mutations in RyR1, as well as acquired channel alterations, are the underlying cause of various skeletal muscle diseases. The aim of this mini review is to summarize several current aspects of RyR1 function, structure, regulation, and to describe the most common diseases caused by hereditary or acquired RyR1 malfunction.

## Introduction to excitation-contraction coupling

The process of voluntary muscle contraction starts with the intention to move, as generated in the frontal lobe and motor cortical regions of the brain (Dum and Strick, 2002). These cortical areas extend neuronal projections to the spinal cord (Rizzolatti and Luppino, 2001). The motoneurons' dendritic arbors and cell bodies serve as the final processing station for corticospinal signals; their axons then leave the spinal cord forming motor nerves, which subsequently deviate from the nerve as it finally approaches the target muscle. Single axons then branch to innervate and control a fixed number of muscle fibers, collectively known as the motor unit. The action potential ultimately reaches the neuromuscular junction, a specialized synapse between the motoneuron and its target muscle fiber, where acetylcholine (ACh) is released from the presynaptic membrane (Hubbard, 1973; Katz, 1996) and binds to postsynaptic ACh nicotinic receptors (Marques et al., 2000; Wu et al., 2010). Activation of ACh nicotinic receptors induces an end-plate potential and this electrical impulse continues along the muscle fiber as an action potential. The process by which this electrical impulse of muscle fibers (aka myofibers) initiates muscle contraction is termed excitation-contraction coupling (ECC; Sandow, 1952).

**Figure 1:**
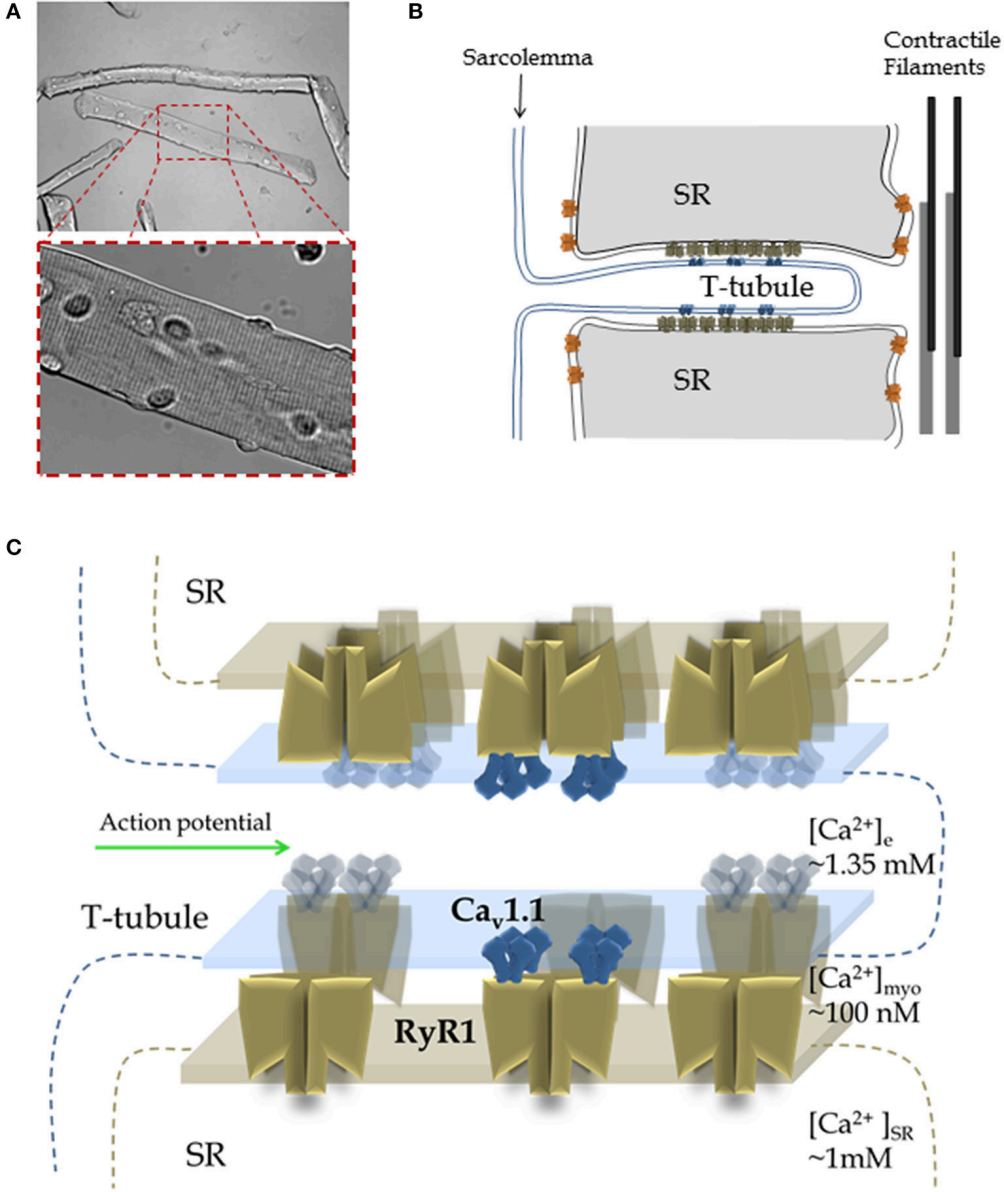
**Muscle structure and function: from the myofiber to RyR1 Ca^2+^ release complex**. **(A)** Morphology of skeletal muscle fibers. Top Panel: Transmitted light image of isolated muscle fibers from mouse flexor digitorum brevis via enzymatic dissociation; image was acquired using low magnification. Lower Panel: A higher magnification image of the fiber segment enclosed by a dashed rectangle. Note the characteristic striated pattern of muscle fibers, which results from highly organized array between sarcolemma, sarcoplasmic reticulum (SR), contractile elements and cytoarchitecture of the fibers. **(B)** Structure of the triad. In most cases the myoplasmic side of the junctional SR is closely apposed either to T-tubule or surface membrane, forming different junctions called dyads, triads, or peripheral coupling. The cartoon depicts a longitudinal section of the T-tubule axis, but a cross-section of the triad, a specialized array formed by the T-tubule and two segments of the terminal junctional SR (aka the terminal cisternae). The T-tubules are invaginations of the sarcolemma that propagate the action potential and possess the Ca_v_1.1 voltage sensors that initiate the early steps of ECC. The voltage dependent Ca^2+^ channels, Cav1.1 (blue, aka DHPR), are positioned in both the T-tubule and sarcolemma. The SR Ca^2+^ release channel, RyR1 (brown), is located on the junctional domain of the SR surface, facing the T-tubules, and is also known as the junctional SR face membrane. Some RyRs may be present in adjacent parajunctional SR domains (orange). **(C)** Detailed architecture of the Cav1.1-RyR1 complex shown in **(B)**. About half of the total RyR1s do not associate with Cav1.1, resulting in an alternating pattern of “free” and Cav1.1-associated RyR1s. Note: In addition to Cav1.1 and RyR1, many other proteins form part of the T-tubule- junctional SR complex (e.g., FKPB12, triadin, junctin, Casq1) and are not shown here. Panels **(B,C)** are based on references: (Franzini-Armstrong and Porter, 1964; Franzini-Armstrong and Nunzi, 1983; Block et al., 1988; Franzini-Armstrong and Jorgensen, 1994; Franzini-Armstrong and Kish, 1995; Franzini-Armstrong and Protasi, 1997).

Each action potential is propagated both axially along the myofiber and radially into the myofiber (Figures 1A,B; Huxley and Taylor, 1958; Adrian et al., 1969; Adrian and Peachey, 1973). The action potential depolarization is generated primarily by current through Na_v_1.4 sodium channels, the skeletal muscle voltage-gated sodium channel, localized at the sarcolemma and through the transverse (T)-tubule system of the myofiber (Figure 1B). T-tubules are extensions of the sarcolemma that form a branched network of radial invaginations of the surface membrane, penetrating the myofiber to surround each myofibril, this occurs at the level of the A-I junction in each mammalian muscle sarcomere (Adrian et al., 1970; Adrian and Marshall, 1977; Jurkat-Rott et al., 2006). Depolarization of the T-tubule membrane induces conformational changes in the T-tubule EC coupling voltage sensor, the voltage-gated Ca^2+^ channel (Ca_v_1.1; Figures 1B,C), also known as the dihydropyridine receptor (DHPR; Rios and Brum, 1987). In skeletal muscle, the DHPR is mechanically coupled to the ryanodine receptor Ca^2+^ release channel type 1 (RyR1), which in contrast to the DHPR, rests in the sarcoplasmic reticulum (SR; Figures 1B,C; Takeshima et al., 1989). The Ca_v_1.1-coupled RyR1 channels mediate, by a mechanism poorly understood, rapid Ca^2+^ release from the SR into the cytosol in response to the muscle action potential in the T-tubules, leading to Ca^2+^ binding to thin filament troponin C and activation for contraction (Schneider and Chandler, 1973; Block et al., 1988). The activity of RyR1 is also regulated by Ca^2+^ (Meissner et al., 1986; Endo, 2006), therefore Ca^2+^ released through molecularly coupled RyR1 could activate Ca_v_1.1-uncoupled RyR1s in close proximity by a process known as Ca^2+^-induced Ca^2+^ release (CICR; Endo, 2009) or via allosteric interactions between adjacent RyR1s (Marx et al., 1998). The contribution of CICR and allosteric coupled RyR1 gating during physiological skeletal muscle activity remains controversial (Endo, 2009; Figueroa et al., 2012). In the process of muscle activation, the sequence of communication from Ca_v_1.1 to RyR is called orthograde signaling (Beam and Horowicz, 2004). The opposite sequence of coupling, from RyR1 to Ca_v_1.1 is known as retrograde signaling (Nakai et al., 1996; Dirksen, 2002). Retrograde signaling causes an enhancement in the activity of Ca_v_1.1 in response to the activation of RyR1 induced by Ca_v_1.1 (Dirksen, 2002). Membrane depolarization of the sarcolemma and T-tubule system also activates Ca^2+^ influx through Ca_v_1.1 (Stanfield, 1977; Sanchez and Stefani, 1978). While this Ca_v_1.1-dependent Ca^2+^ flux appears to be important for activation of Ca^2+^-dependent signaling pathways, SR refilling during sustained activity and modulation of energy utilization, it is not essential for active ECC in adult skeletal muscle (Georgiou et al., 2015; Lee et al., 2015).

Ca^2+^ release is subsequently decreased during depolarization by Ca^2+^-induced inactivation of RyR1, a negative feedback mechanism, and eventually terminated by membrane repolarization, which drives the return of Ca_v_1.1 to a closed and resting state by reversing the activation of the Ca_v_1.1 voltage sensor (Schneider and Hernández-Ochoa, 2012). Muscle relaxation then results from Ca^2+^ removal from myoplasm via transport back to the SR primarily via sarcoplasmic-endoplasmic reticulum ATPase (SERCA), the SR ATP-dependent Ca^2+^ pump (Hasselbach, 1964; Schneider and Simon, 1988; Schneider and Hernández-Ochoa, 2012). These processes prevent continuous Ca^2+^ influx, restore the initial resting state, and allow the ECC cycle to be repeated (Melzer et al., 1995; Berchtold et al., 2000).

## RyR1 function, structure, and regulation

**Figure 2:**
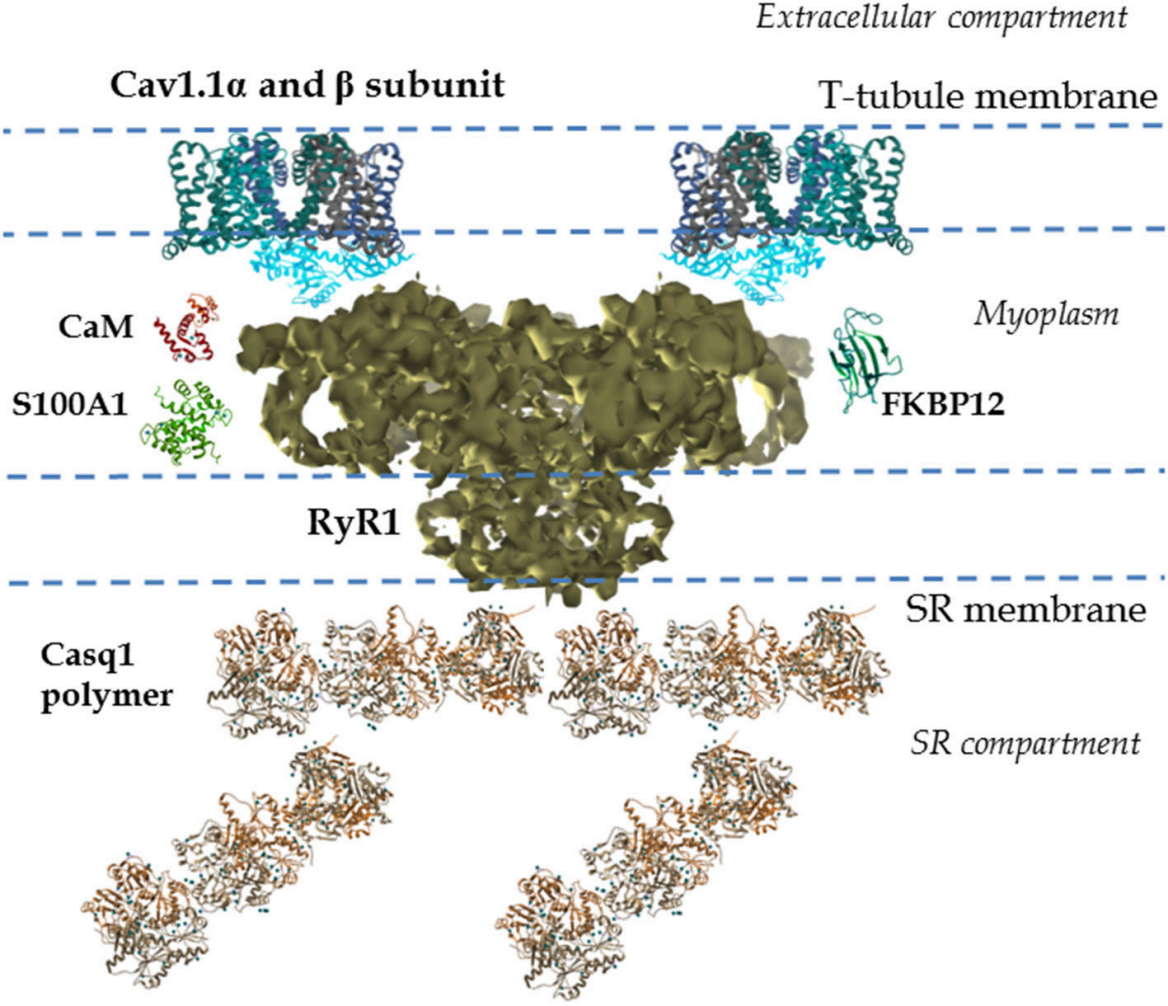
**Cav1.1-RyR1 complex: molecular details**. Shown is a reconstruction from cryo-electron microscopy of the mammalian RyR1 at 4.8 Å illustrating the different regions. This includes the large myoplasmic segment and its multiple cavities and processes, and the SR transmembranal segment, which forms the Ca^2+^ conduction pathway (Electron microscopy data bank entry 6106, Zalk et al., 2015). The above properties allow the RyR1 to interact with multiple SR luminal and cytosolic proteins. Several 3-D structures of proteins interacting with RyR1, as well as their relative locations, are also shown. EMData bank PDB entry and references: calsequestrin-1 (Casq1) polymer (3UOM, Sanchez et al., 2012), FKBP-12 (1D6O, Burkhard et al., 2000), S100A1 (2K2F; Wright et al., 2008), calmodulin (CaM; 1CLL, Chattopadhyaya et al., 1992), Cav1.1α subunit (4MS2, Tang et al., 2014; Hu et al., 2015), and Cav1.1 β subunit (1T0J, Van Petegem et al., 2004; Hu et al., 2015); only two alpha and two beta subunits are shown. In the muscle fiber Ca_v_1.1 channels are clustered in groups of four (or tetrads) in the T-tubules. Ca_v_1.1 channels in the tetrad interact with the four subunits of the RyR1, one Cav1.1 per each RyR1 subunit. All structures were generated using UCSF Chimera (Pettersen et al., 2004).

The function of skeletal muscle relies on the movement of Ca^2+^ out of and back into the storage compartment, the SR (Green and MacLennan, 2002). Release of Ca^2+^ from the SR, via RyR1, results in muscle contraction. The RyR1 is a ~2.3 MDa assembly of four identical subunits (each subunit is formed by ~5000 amino acids; Imagawa et al., 1987; Inui et al., 1987). The subunit topology consists of a pore region formed by six transmembrane helices located within the C-terminal region, representing ca. 20% of total protein. The N-terminal region is a large cytoplasmic region that represents 80% of the total protein, known as the foot region (see Figure 2; Radermacher et al., 1992; Serysheva et al., 1995, 2005, 2008; Ludtke et al., 2005; Samso et al., 2005, 2006, 2009; Yan et al., 2015; Zalk et al., 2015). The myoplasmic portion of the channel (280 Å × 280 Å × 120 Å) is continuous with the transmembrane region (120 Å × 120 Å × 60 Å; Figure 2). The SR transmembranal region forms the Ca^2+^ release channel (Ludtke et al., 2005; Samso et al., 2005, 2006; Serysheva et al., 2005; Yan et al., 2015; Zalk et al., 2015).

RyR1s are organized in regular checkered arrays within the terminal cisternae of the junctional SR (Franzini-Armstrong and Nunzi, 1983; Franzini-Armstrong et al., 1998). Ca_v_1.1 channels are clustered in groups of four (or tetrads) in the T-tubule membrane portion that is in the vicinity of junctional SR (Block et al., 1988). Ca_v_1.1 channels in the tetrad assume a coordinated location relative to the four subunits of the apposed RyR1 (see Figure 1C; Block et al., 1988), however these interactions only occur at every other RyR1. This results in half of RyR1s that are not associated with Ca_v_1.1 (uncoupled RyR1; Franzini-Armstrong and Kish, 1995). The location of coupled and uncoupled RyR1 is dictated by the organization of the Ca_v_1.1 molecules in the apposed T-tubule membrane, and thus mark a “checkerboard” array of coupled and uncoupled RyR1s that follows the Cav1.1 lattice organization (see Figure 1; Yin and Lai, 2000). Depolarization-induced activation of RyR1 is mediated via direct interactions with T-tubule voltage-sensitive Ca_v_1.1 channels positioned in the abutted T-tubule membrane (Rios and Brum, 1987; Nakai et al., 1998a,b; Protasi et al., 2002). Exactly where and how these T-tubule voltage-dependent interactions between Ca_v_1.1 and RyR1 occur remains elusive.

The RyR1 components, including SR luminal segments, transmembrane domains, large cytosolic domains, and interaction with the Ca_v_1.1 channels, allow the RyR1s to be modulated and fine-tuned by multiple mechanisms (Serysheva et al., 2005; Samso et al., 2006; Yan et al., 2015; Zalk et al., 2015). Intracellular ions and components (e.g., cytosolic Ca^2+^, ATP, Mg^2+^, SR Ca^2+^ concentration, Meissner et al., 1986; Fill and Copello, 2002) and post-translational modifications (e.g., oxidation, S-nitrosylation, phosphorylation, Sonnleitner et al., 1997; Marengo et al., 1998; Eu et al., 2000; Sun et al., 2001; Fill and Copello, 2002; Aracena et al., 2003; Hidalgo et al., 2005) all regulate the activity of the RyR1. The RyR1 also interacts with numerous SR intraluminal proteins (e.g., calsequestrin, junctin, triadin; MacLennan and Wong, 1971; Brillantes et al., 1994; Glover et al., 2002; Beard et al., 2009; Treves et al., 2009), cytosolic proteins (e.g., FKBP12, calmodulin (CaM), S100A1; Fuentes et al., 1994; Buratti et al., 1995; Ikemoto et al., 1995; Tripathy et al., 1995; Ahern et al., 1997; Samso and Wagenknecht, 2002; Chelu et al., 2004; Samso et al., 2006; Prosser et al., 2008; Wright et al., 2009) and domains of the Ca_v_1.1 α and β subunits (Nakai et al., 1994, 1998a,b; Gregg et al., 1996; Beurg et al., 1999a,b; Sheridan et al., 2006), located in the T-tubule and myoplasm, respectively. Further details on RyR1 structure-function relationship can be found in recent literature (Van Petegem et al., 2004; Lanner et al., 2010; Lanner, 2012; Van Petegem, 2015).

Given the critical role of RyR1s for skeletal muscle ECC, mutations in the RyR1s and the resulting Ca^2+^ dysregulation are a primary cause of several hereditary skeletal muscle myopathies. In addition, indirect or secondary RyR1 malfunction is also present in other myopathies not related to RyR1 mutations. Both congenital and acquired myopathies are a heterogeneous group of skeletal muscle weakness disorders caused by mutations or dysfunction in different structural, contractile, or regulatory muscle proteins. Several reviews have been published on various aspects of skeletal muscle myopathies (Betzenhauser and Marks, 2010; Lanner et al., 2010; Maclennan and Zvaritch, 2011; Lanner, 2012; Dowling et al., 2014; Vallejo-Illarramendi et al., 2014). Here we focus on primary RyR1 mutation-related myopathies, as well as considering Duchenne muscular dystrophy (DMD), a disease with a secondary involvement of RyR1.

## RyR1 primary mutation-related myopathies

### General implication of RyR1 disease mutations

Mutations in RyR1 can be grouped into four general categories according to their effects on RyR1 function (Treves et al., 2008; Loy et al., 2011). The first category of RyR1 mutations cause a higher probability of activation of RyR1 by muscle fiber electrical depolarization or by RyR1 activators, and are manifested in the malignant hyperthermia (MH) phenotype. In the second category, RyR1 mutations cause leaky channels leading to Ca^2+^ dysregulation and depletion of Ca^2+^ from SR, and result in central core disease (CCD). The third category of RyR1 mutations causes deficits on the Ca_v_1.1-mediated voltage dependent activation of SR Ca^2+^ release, a process also known as excitation-contraction uncoupling, and result in certain forms of CCD. The fourth category of mutations are a consequence of wild type allele silencing, a process that mimics homozygosity, and causes a decrease of RyR1 channel expression, resulting in multi-minicore disease (MmD.) In the majority of these primary myopathies, mutations in the RyR1, result in gain of function and Ca^2+^ dysregulation. The consequences of these mutations on RyR1's activity are compound; RYR1-related MH and CCD produce hypersensitive and/or leaky RyR1 channels (Tong et al., 1997; Lynch et al., 1999; Yang et al., 2007). Some CCD present excitation-contraction uncoupling (Avila et al., 2001; Dirksen and Avila, 2002; Kraeva et al., 2013) or exhibit RyR1 leaky channels (Dirksen and Avila, 2002; Zvaritch et al., 2009), but the exact mechanisms are unclear (Dowling et al., 2014). Many RyR1 mutations are not so obviously correlated with alterations in Ca^2+^ release (Maclennan and Zvaritch, 2011; Dowling et al., 2014), and their pathogenicity is less clear.

### Malignant hyperthermia

Malignant hyperthermia is a subclinical, autosomal dominant, pharmacogenetic disorder that causes skeletal muscle intracellular Ca^2+^ dysregulation, characterized by a sudden and potentially fatal hypermetabolic adverse response to volatile anesthetics (e.g., isoflurane, sevoflurane, halothane) and/or the depolarizing muscle relaxant, succinylcholine (Denborough et al., 1962; Rosenberg et al., 2007; Maclennan and Zvaritch, 2011). MH manifests mostly in individuals carrying mutations in RyR1 (MacLennan et al., 1990), but some Ca_v_1.1 mutations are also linked to MH (Rosenberg et al., 2007; Maclennan and Zvaritch, 2011). When MH-susceptible individuals are expose to trigger agent(s) (Denborough et al., 1962), they develop hypercarbia, tachycardia, muscle rigidity (some cases), rapid and severe hyperthermia (e.g., core body temperatures of 43°C), rhabdomyolysis and metabolic acidosis (Rosenberg et al., 2007; Larach et al., 2010; Maclennan and Zvaritch, 2011). MH may occur at any time during the anesthesia and in the postoperative period (Rosenberg et al., 2007). MH episodes account for many anesthetic-induced deaths in the operating room in otherwise healthy individuals (Maclennan and Zvaritch, 2011; Rosenberg et al., 2007). Untreated, episodes of MH are lethal in 90% of the cases. Consequences of MH include damage to brain, kidney and muscle tissues. In United States, MH complicates 1 out of 100,000 surgeries in adults and 1 out of 30,000 surgeries in children (Malignant Hyperthermia Association of United Sates; http://www.mhaus.org).

Different regions of RyR1 are targets of MH causing mutations (Manning et al., 1998; Barone et al., 1999; Brandt et al., 1999; Chamley et al., 2000; Rueffert et al., 2004; Betzenhauser and Marks, 2010). Dominant RyR1 mutations are the leading cause of MH. Studies on the Ca^2+^ conducting properties of RyR1 channels containing MH mutations expressed in myotubes (Dietze et al., 2000; Yang et al., 2003; Chelu et al., 2006; Bannister et al., 2010) and mature muscle fibers (Owen et al., 1997; Andronache et al., 2009), as well as animal models of MH (Mickelson and Louis, 1996; Chelu et al., 2006) and affected humans (Denborough et al., 1962; Melzer and Dietze, 2001), have concluded that MH mutations cause a channel gain of function, characterized by hyper-activation and hyper-sensitization to pharmacological activators. Current treatment includes immediate suspension of the triggering agent(s), cooling measures (hypothermic blankets), and 100% oxygen administration, as well as prompt treatment with dantrolene, a drug that acts as Ca^2+^ release inhibitor (Hainaut and Desmedt, 1974; Kobayashi et al., 2005), presumably acting on RyR1 (Fruen et al., 1997) and that requires of CaM for its effects (Oo et al., 2015). Ca^2+^ entry, via non-specific sarcolemmal channels, contributes to the pathogenesis of MH episodes (Eltit et al., 2013). Dantrolene also attenuates elevations on resting [Ca^2+^] that is dependent on Ca^2+^ entry (Cherednichenko et al., 2008; Eltit et al., 2013). Because dantrolene appears to suppress Ca^2+^ release and Ca^2+^ entry, it is not surprising that dantrolene could affect other proteins involved in ECC. Regardless of the action(s), dantrolene is currently the only available pharmacological treatment for MH (Mickelson and Louis, 1996; Lyfenko et al., 2004; Litman and Rosenberg, 2005) and reduces MH mortality significantly (Rosenberg et al., 2007; Schneiderbanger et al., 2014). Susceptibility to MH attacks can be detected prior to surgery using *in-vitro* assays that measure muscle contractile responses and halothane or caffeine sensitivity assays using muscle biopsies from individuals at risk or with family history of enhanced sensitivity to these agents, or in known individuals with MH-mutations (Rosenberg et al., 2007; Schneiderbanger et al., 2014).

### The RyR1-related congenital myopathies: Central core disease and multi-minicore disease

These RyR1-related congenital myopathies are rare muscle disorders (incidence of 6 out of 100,000 births) that exhibit either autosomal dominant or recessive inheritance patterns. They are typically manifested at birth, or during childhood, and are characterized by generalized muscle weakness and low muscle tone (Magee and Shy, 1956; Shuaib et al., 1987; Jungbluth, 2007a,b). There are many forms of congenital myopathy, each defined by a specific pattern of histological abnormalities, and clinical course severity (Jungbluth, 2007a,b; Nance et al., 2012). Most individuals with congenital myopathies follow a relatively stable clinical course. Typically these patients present with ophthalmoparesis (eye muscle weakness), myasthenia (muscle weakness), and abnormal movement due to joint contractures. Patients may develop dysfunction of respiratory muscles, scoliosis, or difficulty swallowing (dysphagia; Jungbluth, 2007a,b). In comparison to MH episodes, the symptoms in CCD are not elicited by an external trigger. Currently, there is no cure for CCD; the treatment is supportive and aimed to maintain mobility and independence (Jungbluth, 2007a,b). At the cellular level, core myopathies are characterized by regions in muscle fibers devoid of mitochondria that appear as “cores” on oxidative stains (Dubowitz and Pearse, 1960; Gonatas et al., 1965). In CCD, cores are large and extend longitudinally. In MmD, the cores are short and vary in size and location (Sewry et al., 2002). Mutations in RYR1 are an important cause/factor of several forms of congenital myopathies, >60 mutations in RyR1 are linked to CCD (Quane et al., 1993; Zhang et al., 1993; Treves et al., 2008). Studies on the Ca^2+^ conducting properties of RyR1 channels containing CCD mutations expressed in myotubes concluded that CCD mutations cause SR Ca^2+^ leak due to hyperactive RyR1 channels (Tong et al., 1997; Lynch et al., 1999) or ECC uncoupling (Avila et al., 2001; Dirksen and Avila, 2002), and link these events to the development of cores and muscle weakness. The specific underlying process that causes the core formation is still unknown.

### Secondary involvement of RyR1 in muscle Duchenne muscular dystrophy

DMD, a devastating and lethal inherited X-linked neuromuscular disease, is one of the most common genetic diseases (Mah et al., 2014). DMD almost exclusively affects boys, resulting in muscular dysfunction and premature death (Emery, 2002). The primary defect in DMD is the lack of dystrophin, a 427 kDa cytoskeletal protein that structurally links the contractile machinery to the dystrophin-glycoprotein complex and to components of the extracellular matrix (Hoffman et al., 1987; Campbell, 1995; Blake et al., 2002). Lack of dystrophin results in myofibers structurally defective and prone to damage. However, the absence of dystrophin alone cannot immediately account for the intricate clinical course and pathogenic mechanisms seen in DMD (Berchtold et al., 2000; Allen et al., 2010; Allen and Whitehead, 2011), and multiple hypotheses have been proposed as intervening events to explain the pathological alterations observed in DMD (Florence et al., 1985; McArdle et al., 1995; Tutdibi et al., 1999; Berchtold et al., 2000; Lovering et al., 2009a, 2013; Allen et al., 2010; Allen and Whitehead, 2011; Pratt et al., 2013, 2015; Xu et al., 2012; Mazala et al., 2015).

Regarding the involvement of RyR1 in muscular dystrophy, it is well established that dystrophin-deficient myofibers, especially those with malformed morphology (Lovering et al., 2009b; Hernández-Ochoa et al., 2015), have deficits in ECC (Collet et al., 1999; Woods et al., 2004, 2005; Hollingworth et al., 2008; Lovering et al., 2009b; Hernández-Ochoa et al., 2015), which is manifested as altered action potential-elicited RyR1 Ca^2+^ release from the SR (Woods et al., 2004). Several proposed mechanisms might explain the differences in ECC found between healthy and dystrophin-deficient myofibers, among them are alterations in the expression levels of Ca_v_1.1 and RyR1, changes in Ca^2+^ buffering capacity (Doran et al., 2004; Dowling et al., 2004), dysfunction of membrane channels (Yeung et al., 2005), and differences in levels of ROS (Whitehead et al., 2010; Allen and Whitehead, 2011).

There is also evidence that increased S-nitrosylation of RyR1 in dystrophin deficient muscles, leads to FKBP12-RYR1 destabilization and increases RyR1 Ca^2+^ leak (channel opening at rest; Bellinger et al., 2009). It is likely that these modifications occur at intermediate or end stages of the transition from normal to apoptotic/necrotic fiber, where RyR1 leak will worsen Ca^2+^ dysregulation.

Thus, DMD appears to affect the activity of the RyR1 in two opposite ways: (1) First, in early stages of myofiber damage, or in pre-apoptotic phases, the overall function of RyR1 is reduced, leading to depressed action potential-induced RyR1 Ca^2+^ release (Collet et al., 1999; Woods et al., 2004, 2005; Hollingworth et al., 2008; Lovering et al., 2009b; Hernández-Ochoa et al., 2015). Since the magnitude of myofiber contraction and consequent force production are finely controlled by intracellular Ca^2+^ release (Chin, 2010), in DMD a gradual reduction RyR1 Ca^2+^ release contributes to the development of muscle weakness. (2) In advanced stages of the disease, RyR1 becomes hypersensitive to intracellular Ca^2+^ activation. Functional and post-translational modifications in RyR1 contribute to altered Ca^2+^ homeostasis in dystrophin-deficient muscles (Bellinger et al., 2009; Allen et al., 2010; Allen and Whitehead, 2011). Age-dependent S-nitrosylation of the RyR1 complex causes depletion of FKBP12 from the RyR1, resulting in a remodeling of the RyR1 channel complex, which in turn elicits intracellular SR Ca^2+^ leak and impaired contractility (Bellinger et al., 2009). The concurrent increased Ca^2+^ influx across the plasma membrane, due to inherent membrane fragility and consequent membrane damage, is thought to cause further membrane damage via multiple mechanisms including calpain activation (Berchtold et al., 2000; Allen et al., 2010; Allen and Whitehead, 2011).

Despite recent advances in understanding DMD, targeted pharmacological treatment for DMD is nearly non-existent (Andersson and Marks, 2010). In this regard, a new class of small-molecules “Rycals,” which reduce Ca^2+^ leak by stabilizing the FKBP12-RyR1 interaction, have been shown to improve contractility in skeletal muscle in the mdx mouse model of DMD (Bellinger et al., 2009). Rycal ARM210, has potential as a treatment for DMD; it has completed preclinical efficacy and safety studies, and has been selected for advancement to clinical development (Muscle Dystrophy Association; https://www.mda.org).

Given the critical role of RyR1 in Ca^2+^ release and Ca^2+^ signaling, any disease condition that affects RyR1 function via post-translational modifications could affect ECC, Ca^2+^-dependent metabolic and genetic programs, and untimely impinge on skeletal muscle function. In support of this view, recent reports have shown post-transcriptional alterations of RyR1 in muscle weakness related to arthritis (Yamada et al., 2016) and cancer (Waning et al., 2015) in murine models.

## Conclusions

Many skeletal myopathies result from intracellular Ca^2+^ dysregulation and alterations in a myriad of Ca^2+^ dependent processes, including muscle contraction, signaling pathways, metabolism and gene regulation. Reduced SR Ca^2+^ release translates to deficient muscle contraction and muscle weakness. Sustained increases in cytoplasmic [Ca^2+^] or decreased SR intraluminal Ca^2+^ are key pathological events leading to muscle contractures (i.e., in MH episodes), apoptosis and necrotic muscle degeneration (i.e., dystrophinopathies; Maclennan and Zvaritch, 2011). In addition, many key channels, transporters, Ca^2+^ buffers and modulators of Ca^2+^ signaling contribute to abnormal Ca^2+^ handling in several muscle myopathies. Skeletal muscle research will continue to expand our understanding of these myopathies, as well as the function and dysfunction of the RyR1. Despite that the current near-atomic resolution RyR1 reconstructions are in the 4.8–3.8 Å range, more detailed RyR1 images are still needed, as well as specifics about RyR1's interactions with Ca_v_1.1 and many other auxiliary and modulatory proteins (Van Petegem, 2015). Also, more in-depth function-structure studies (from molecular biophysics to systems physiology), increased sensitivity of structural biology methods and likely the development of new biochemical and physiological assays, are required. These studies will propel our knowledge about RyR1 function and dysfunction, and facilitate the development of new RyR1-selective drugs for RyR1-related myopathies.

## Author contributions

All authors contributed to this paper with literature review, manuscript drafting, editing, and final approval of the final version.

## Funding

Grants to MS (R37-AR055099) and to RL (R01AR059179) from National Institutes of Health (NIH), National Institute of Arthritis and Musculoskeletal and Skin Diseases. The content is solely the responsibility of the authors and does not necessarily represent the official views of the NIH.

### Conflict of interest statement

The authors declare that the research was conducted in the absence of any commercial or financial relationships that could be construed as a potential conflict of interest.
